# Metabolic engineering of *Escherichia coli* for the utilization of ethanol

**DOI:** 10.1186/s40709-020-0111-0

**Published:** 2020-01-21

**Authors:** Yujin Cao, Hui Mu, Jing Guo, Hui Liu, Rubing Zhang, Wei Liu, Mo Xian, Huizhou Liu

**Affiliations:** 1 CAS Key Laboratory of Biobased Materials, Qingdao Institute of Bioenergy and Bioprocess Technology, Chinese Academy of Sciences, Qingdao, 266101 China; 2 Energy Research Institute, Shandong Key Laboratory of Biomass Gasification Technology, Qilu University of Technology (Shandong Academy of Sciences), Jinan, China

**Keywords:** Alcohol dehydrogenase, Aldehyde dehydrogenase, Ethanol utilization, *Escherichia coli*, Mevalonic acid

## Abstract

**Background:**

The fuel ethanol industry has made tremendous progress in the last decades. Ethanol can be obtained by fermentation using a variety of biomass materials as the feedstocks. However, few studies have been conducted on ethanol utilization by microorganisms. The price of petroleum-derived ethanol, easily made by the hydrolysis of ethylene, is even lower than that of bioethanol. If ethanol can be metabolized by microorganisms to produce value-added chemicals, it will open a new door for the utilization of inexpensive ethanol resources.

**Results:**

We constructed an engineered *Escherichia coli* strain which could utilize ethanol as the sole carbon source. The alcohol dehydrogenase and aldehyde dehydrogenase from *Aspergillus nidulans* was introduced into *E. coli* and the recombinant strain acquired the ability to grow on ethanol. Cell growth continued when ethanol was supplied after glucose starvation and 2.24 g L^−1^ of ethanol was further consumed during the shake-flasks fermentation process. Then ethanol was further used for the production of mevalonic acid by heterologously expressing its biosynthetic pathway. Deuterium-labeled ethanol-D6 as the feedstock confirmed that mevalonic acid was synthesized from ethanol.

**Conclusions:**

This study demonstrated the possibility of using ethanol as the carbon source by engineered *E. coli* strains. It can serve as the basis for the construction of more robust strains in the future though the catabolic capacity of ethanol should be further improved.

## Background

Ethanol is a simple alcoholic compound with the chemical formula of C_2_H_6_O and has a variety of applications in chemical, food, medical and health industries. It is commonly used in beverages, flavors, fuels, dyes as well as manufacturing disinfectant, antifreeze and paint. In addition to the non-renewable chemical process to be made from petroleum, coal and natural gas, ethanol can also be produced from biomass by fermentation of sugars, starch or cellulose as raw materials [1]. Microorganisms including bacteria, yeasts and fungi which can produce ethanol as the major fermentation product have been extensively studied [2]. Many of them were engineered to improve their ethanol production capacity [3]. On the other hand, the utilization of ethanol by microorganisms has not yet been thoroughly investigated, though ethanol metabolic pathways are ubiquitous in nature.

Several microbial strains were identified to be capable of utilizing ethanol. It is well known that the acetic acid bacteria can oxidize ethanol to acetic acid [4]. But whether ethanol could serve as the carbon and energy source for these bacteria was not clearly established. *Acinetobacter baylyi* was able to produce storage lipids using different carbon sources and ethanol showed the highest specific growth rates among them [5]. *Methanogenium organophilum*, a non-autotrophic methanogenic bacterium, was able to use ethanol as the hydrogen donor [6]. The sulfate-reducing bacterium *Desulfovibrio desulfuricans* was tested using ethanol as the carbon source. Growth yield was lower for ethanol in comparison with lactic acid [7]. The yeasts *Pichia pastoris* [8], *Candida utilis* [9] and *Yarrowia lipolytica* [10] could utilize ethanol as the carbon source and eventually convert it into biomass and metabolites. However, this ability was not studied in detail and the mechanisms for ethanol oxidation in these strains were still unclear.

**Fig. 1:**
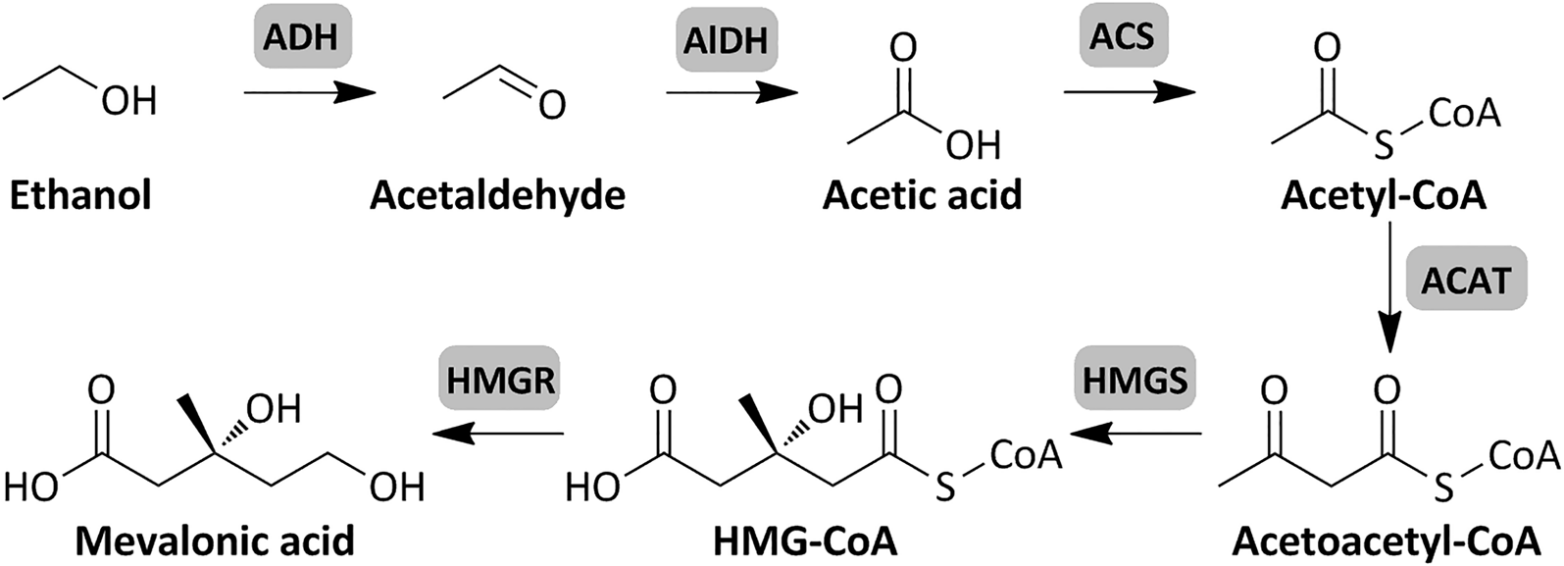
Metabolic pathway for ethanol utilization and mevalonic acid production. Enzymes involved in the pathway include: *ADH* alcohol dehydrogenase, *ALDH* aldehyde dehydrogenase, *ACS* acetyl-CoA synthetase, *ACAT* acetyl-CoA acetyltransferase, *HMGS* HMG-CoA (3-hydroxy-3-methylglutaryl CoA) synthase, *HMGR* HMG-CoA reductase

Although the above microorganisms could utilize ethanol, they are not easily genetically modified to produce high-value chemicals. *Escherichia coli* is one of the most commonly used host microorganisms in industrial biotechnology [11]. In early reports, it was found that ethanol disappeared from cultures of *E. coli* under anaerobic conditions [12]. Further ^14^C isotope labeling experiments showed that ethanol was incorporated into the cell components when *E. coli* grew on a synthetic medium [13]. However, there has been no further study since then. Ethanol catabolism pathway in *Aspergillus nidulans* has been well characterized [14]. Ethanol is oxidized by alcohol dehydrogenases to generate acetaldehyde. Acetaldehyde is converted to acetic acid by aldehyde dehydrogenases. Acetic acid is then activated by an acetyl-CoA synthetase and the formed acetyl-CoA is used for the synthesis of other metabolites (Fig. 1). In this study, different alcohol dehydrogenases and aldehyde dehydrogenases were expressed in *E. coli* to test their feasibility for ethanol utilization. Furthermore, an acetyl-CoA derived metabolite-mevalonic acid biosynthesis pathway was introduced into the engineered strain to demonstrate the potential of value-added product production from ethanol.

## Results

### *Escherichia coli* strains cannot utilize ethanol as the sole carbon source

Ethanol is a mild solvent and causes less damage to microbial cells. Several *E. coli* strains commonly used in laboratory, including BL21(DE3), DH5α, Top10 and JM109 which differ in the coding regions of a few genes, were used to evaluate their ethanol resistance and adaptation. These strains were grown in M9 mineral media supplemented with 20 g L^−1^ glucose and 1 mM MgSO_4_, and then treated with ethanol of different concentrations (5 g L^−1^, 10 g L^−1^, 20 g L^−1^ and 50 g L^−1^). We found that ethanol with a concentration as high as 50 g L^−1^ did not significantly affect the growth of all the *E. coli* strain (data not shown). Although the presence of ethanol could alter the gene expression pattern of *E. coli* [15], it does not have serious effects on normal cell metabolism. Ethanol can be directly added to the culture broth at relatively high concentrations.

**Fig. 2:**
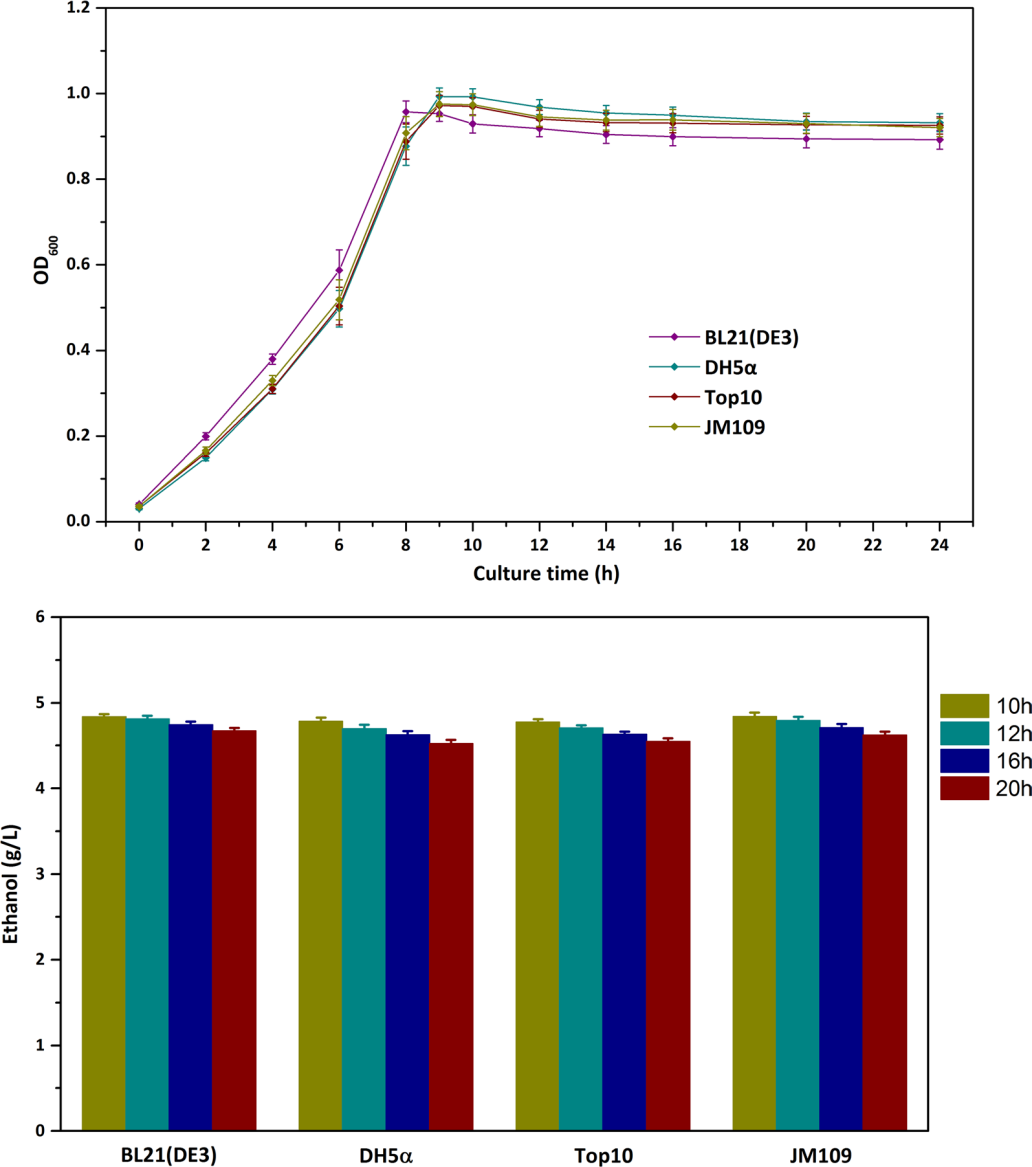
Growth curves and ethanol utilization of commonly used *E. coli* strains. **a** Growth curves of strains BL21(DE3), DH5α, Top10 and JM109; **b** residual ethanol concentrations in the fermentation broth of strains BL21(DE3), DH5α, Top10 and JM109

In order to test the feasibility of *E. coli* to grow on ethanol, these strains were cultured in M9 mineral media with reduced glucose concentration of 1 g L^−1^. After the initial glucose was exhausted (about 8 h for strain BL21(DE3) and 9 h for the other three strains), 5 g L^−1^ of ethanol was added to the fermentation broth. The bacterial cells were cultured continuously and cell densities were measured over the whole processes. As shown in Fig. 2a, the growth of all the strains ceased at an OD_600_ of 1.0 after ethanol was supplied. When monitoring ethanol concentrations in the culture broth, we found that the residual ethanol was slightly decreased, which might be due to evaporation during the fermentation process (Fig. 2b). In early literature reports [13], *E. coli* was demonstrated to have the ability to metabolize ethanol. The different genotype between *E. coli* sub-strains might lead to their diverse ethanol utilizing ability. Nowadays, the commercially available *E. coli* K12 derivatives lack many genes to achieve good heredity stability [16], which impairs the ability to metabolize various substrates.

### Construction of the ethanol utilization pathway in *E. coli*

The ethanol utilization pathway requires successive dehydrogenation reactions to form acetic acid. Alcohol/aldehyde dehydrogenases are the key enzymes catalyzing these reactions. *Escherichia coli* genome encodes a variety of alcohol/aldehyde dehydrogenases [17]. Three of them (AdhE, AdhP and AldA) were chosen to evaluate their effects on ethanol utilization. The alcohol/aldehyde dehydrogenase AdhE could naturally catalyze the sequential reduction of acetyl-CoA to acetaldehyde and then to ethanol under fermentative conditions [18]. The expression of alcohol dehydrogenase AdhP was induced by ethanol [19] and AldA has the potential to serve as aldehyde dehydrogenase in the oxidation of aldehyde [20]. The genes encoding these dehydrogenases were cloned into the pTrcHis2B vector to overexpress these enzymes.

The ethanol utilization pathway in the hyphal fungus *Aspergillus nidulans* have been well characterized as an inducible system responsible for the utilization of ethanol as sole carbon source [14, 21]. The action of two enzymes, alcohol dehydrogenase I (ADHI) encoded by the *alcA* gene and aldehyde dehydrogenase (ALDH) encoded by the *aldA* gene was necessary and sufficient for the oxidation of ethanol to acetic acid via acetaldehyde. Therefore, the two structure genes, *alcA* and *aldA*, were also cloned into pTrcHis2B vector individually and simultaneously, resulting pTrc-AnalcA, pTrc-AnaldA and pTrc-AnalcdA. The recombinant plasmids harboring the ethanol utilization pathway were confirmed by colony PCR, restriction enzyme mapping and direct DNA sequencing. Then these recombinant plasmids were transformed into *E. coli* competent cells to evaluate their effects on ethanol utilization.

### Shake-flask fermentation of engineered *E. coli* strains using ethanol as the carbon source

**Fig. 3:**
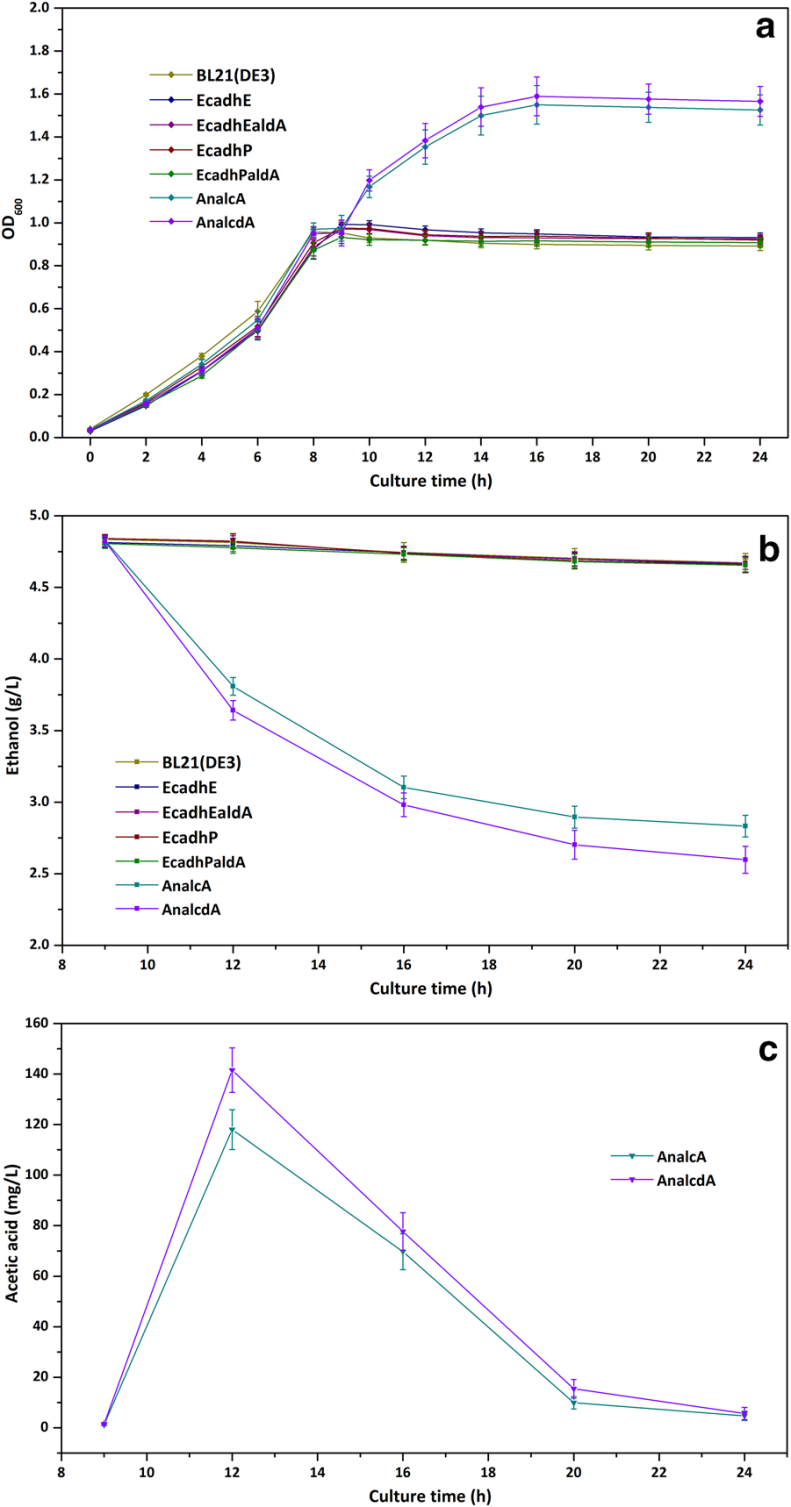
Ethanol utilization of engineered *E. coli* strains with different alcohol/aldehyde dehydrogenases. **a** Growth curves of engineered strains harboring different alcohol/aldehyde dehydrogenases; **b** residual ethanol concentrations of different engineered strains; **c** acetic acid concentrations of strains BL21/pTrc-AnalcA and BL21/pTrc-AnalcdA during the fermentation processes

Recombinant *E. coli* strains expressing different alcohol/aldehyde dehydrogenases were grown on M9 mineral media supplemented with 1 g L^−1^ glucose and 1 mM MgSO_4_. When OD_600_ of the bacterial cultures reached 0.3 or so, 0.2 mM IPTG was added to induce the expression of recombinant enzymes. Then cultivation was continued and residual glucose concentrations were monitored until it was exhausted. The bacterial cells were further starved for 1 h and ethanol was added to the culture broth to serve as the carbon source. As shown in Fig. 3a, strains BL21/pTrc-EcadhE, BL21/pTrc-EcadhP, BL21/pTrc-EcadhEaldA and BL21/pTrc-EcadhPaldA possessed similar growth curves as the wild-type *E. coli* BL21(DE3). Cell growth stopped after the depletion of initial glucose at 8 h after inoculation. AdhE cannot catalyze the reversible reaction of ethanol oxidation. Although the expression of *adhP* was induced by ethanol, the major physiological role of this enzyme is to allow *E. coli* to excrete ethanol during mixed-acid fermentation [22]. The *aldA* gene encoding aldehyde dehydrogenase is mainly responsible for the dehydrogenation of lactaldehyde [20]. Therefore, these enzymes had no effects in the utilization of ethanol. On the contrary, a remarkable increase in OD_600_ was observed for strain BL21/pTrc-AnalcA after ethanol was supplemented in the culture. This indicated that the alcohol dehydrogenase from *A. nidulans*, could readily render *E. coli* the ability to metabolize ethanol and further confirmed that *alcA* was essential for the use of ethanol as the sole carbon source [23]. Obviously, the *alcA* gene was sufficient for ethanol utilization in *E. coli*. When the aldehyde dehydrogenase from *A. nidulans* was co-expressed with *alcA*, a further slight enhancement was achieved in the cell density.

The residual ethanol, acetaldehyde and acetic acid in the fermentation broth were also monitored during the culture process. For strains BL21(DE3), BL21/pTrc-EcadhE, BL21/pTrc-EcadhP, BL21/pTrc-EcadhEaldA and BL21/pTrc-EcadhPaldA, there were only a small decrease in residual ethanol concentrations, which was in accordance with that these strains could not utilize ethanol. For strains BL21/pTrc-AnalcA and BL21/pTrc-AnalcdA, ethanol was consumed immediately after it was added to the flasks. Both strains could utilize more than 2 g L^−1^ ethanol in the remaining cultivation time. The titer of residual ethanol reached 2.60 ± 0.1 g L^−1^ and 2.24 g L^−1^ of ethanol was consumed for the best strain BL21/pTrc-AnalcdA (Fig. 3b). Acetaldehyde accumulation could be hardly detected in all strains. Aldehyde was harmful to bacterial cells. When acetaldehyde was generated by alcohol dehydrogenase, it was rapidly further oxidized to acetic acid by the endogenous aldehyde dehydrogenases [24]. Acetic acid was also not found in the fermentation broth of strains BL21(DE3), BL21/pTrc-EcadhE, BL21/pTrc-EcadhP, BL21/pTrc-EcadhEaldA and BL21/pTrc-EcadhPaldA. On the other hand, both BL21/pTrc-AnalcA and BL21/pTrc-AnalcdA could produce acetic acid after ethanol was supplemented. The maximum titer of acetic acid was 142 ± 9 mg L^−1^ for strain BL21/pTrc-AnalcdA. The acetic acid could be further utilized during the remaining culture time (Fig. 3c).

### Effects of acetyl-CoA synthetase on ethanol utilization

**Fig. 4:**
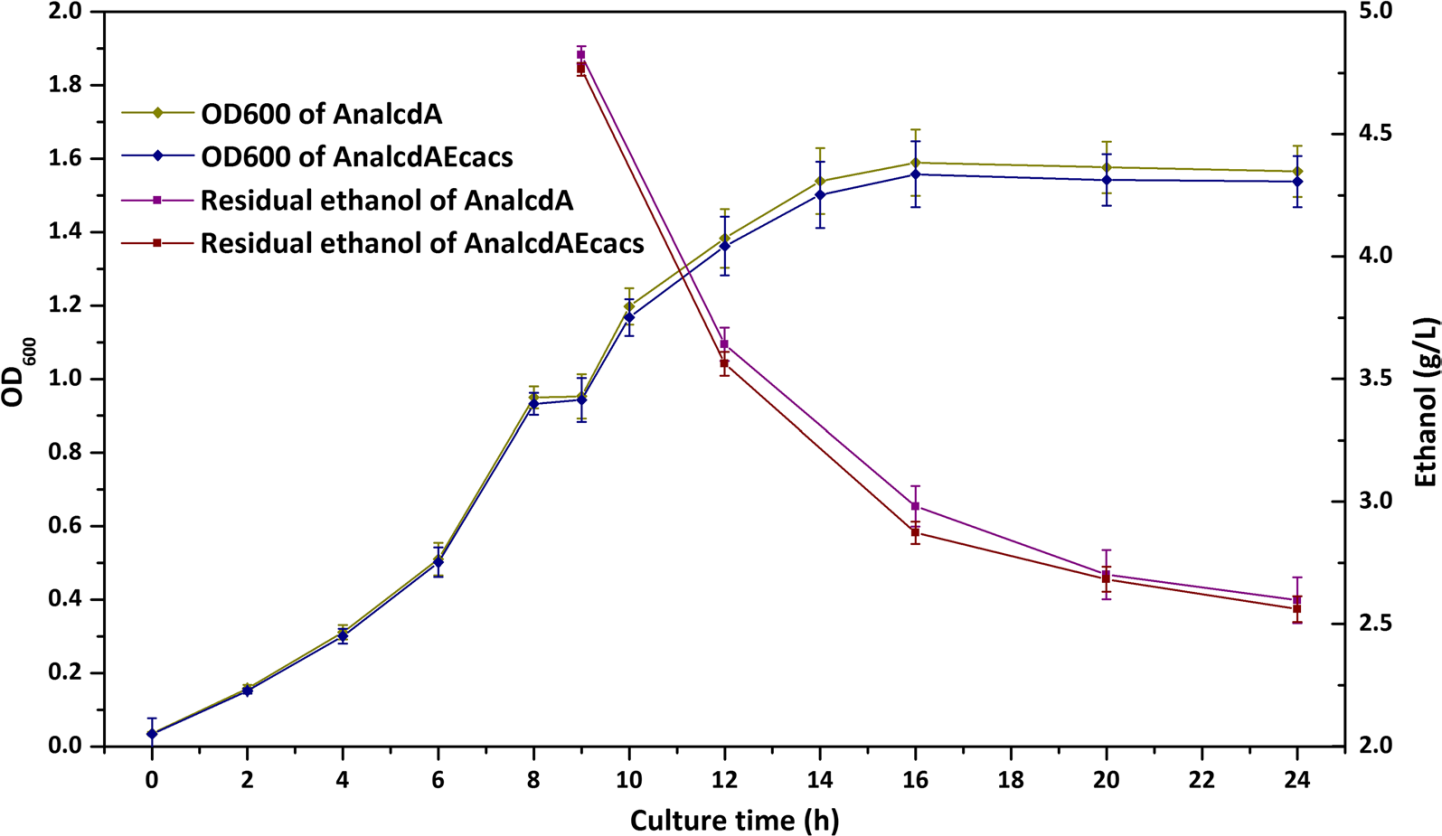
Effects of acetyl-CoA synthetase on ethanol utilization. AnalcdA, *E. coli* BL21(DE3) harboring pTrc-AnalcdA; AnalcdAEcacs, *E. coli* BL21(DE3) harboring pTrc-AnalcdA and pA-Ecacs

Acetyl-CoA synthetase, catalyzing the formation of acetyl-CoA from acetic acid, is the next step for ethanol catabolism. Overexpression of acetyl-CoA synthetase has been demonstrated to enhance the assimilation of acetic acid and the activation to acetyl-CoA, leading to an increase in acetate uptake [25]. Therefore, this enzyme has the potential to enhance ethanol utilization ability of the engineered strain. Here, the native *E. coli* acetyl-CoA synthetase (encoded by *acs*) was cloned into the expression vector pACYCDuet-1, resulting pA-Ecacs. The strain BL21/pTrc-AnalcdA&pA-Ecacs was cultured under the same conditions above. As shown in Fig. 4, there were no significant improvement in cell growth and ethanol utilization. Since acetic acid could only accumulate to no more than 150 mg L^−1^ in the fermentation broth, the normal intracellular level of acetyl-CoA synthetase was enough for ethanol metabolism. In addition, the acetyl-CoA synthetase catalyzed reaction is coupled with ATP hydrolysis, which is an energy consuming process [26]. When using ethanol as the carbon source instead of glucose, ATP generation is much lower. The ATP supply cannot meet the requirement for next-step activation of acetic acid for further metabolism. Therefore, further overexpression of acetyl-CoA synthetase could not enhance ethanol utilization. The dehydrogenation reaction was the rate-limiting step for ethanol catabolism.

### Production of mevalonic acid from ethanol

**Fig. 5:**
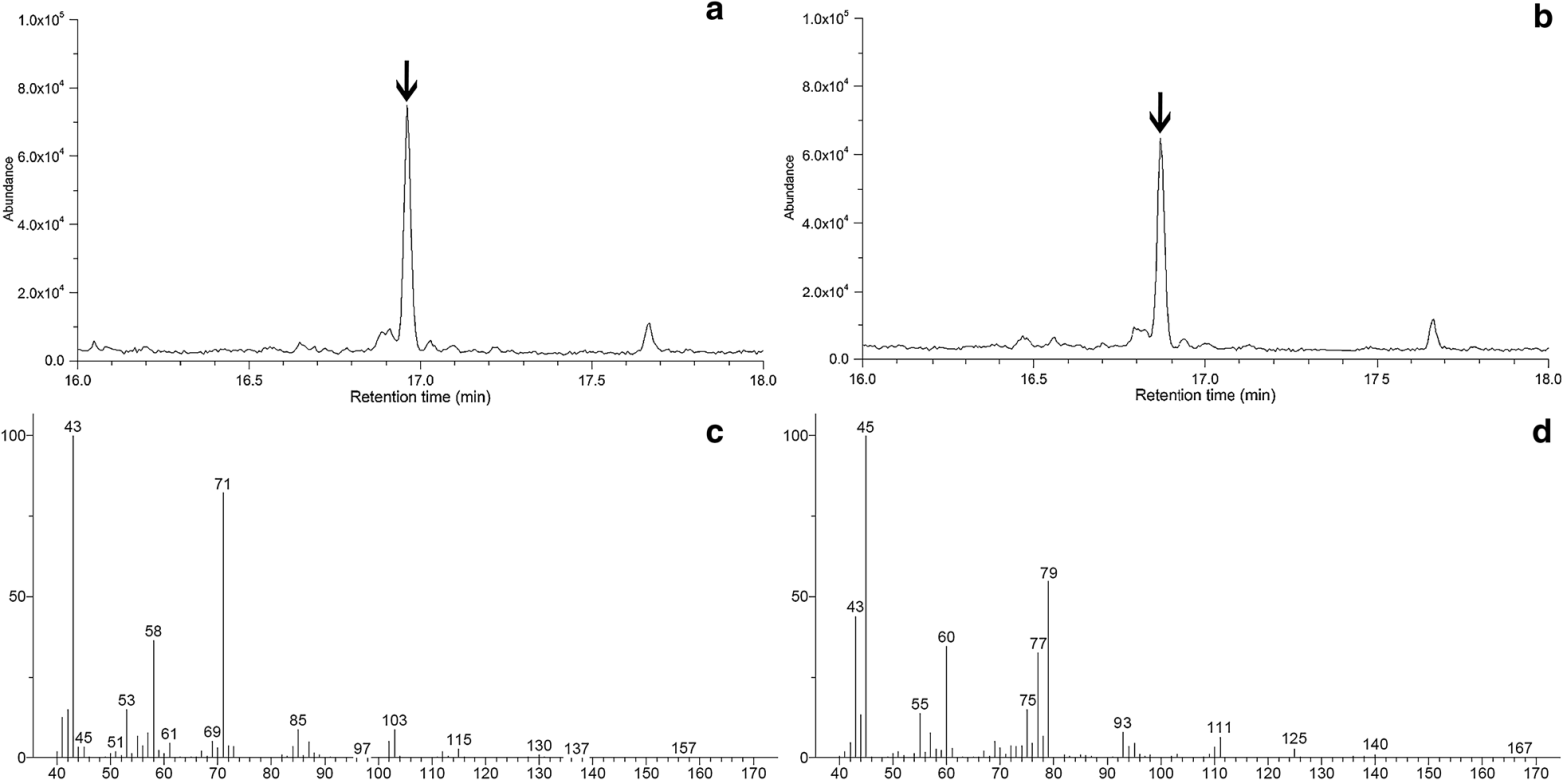
Identification of mevalonic acid lactones by GC–MS. Total ion chromatograms of mevalonic acid lactones produced from ethanol (**a**) and ethanol-D6 (**b**) (the peaks of mevalonic acid lactones were indicated with arrows); mass spectra of mevalonic acid lactones produced from ethanol (**c**) and ethanol-D6 (**d**)

To test the feasibility of using ethanol as the carbon source for value-added chemicals biosynthesis, we further introduced the mevalonic acid biosynthesis pathway previously constructed in our lab into the engineered strain. *Escherichia coli* BL21(DE3) harboring both pTrc-AnalcdA and pA-EfmvaES was cultured in M9 mineral media under shake-flasks conditions. After fermentation, the culture broth was acidified and extracted with ethyl acetate, and then the organic phase was analyzed by GC–MS. Figure 5a showed the total ion current chromatogram of the fermentation products from strain BL21/pTrc-AnalcdA&pA-EfmvaES. The peak corresponding to the retention time of 16.97 min was identified to be mevalonic acid lactone by comparison the mass spectrum (Fig. 5c) with an external standard. The production of mevalonic acid was much lower than the *E. coli* strains expressing the same enzymes cultured using glucose as the carbon source [27]. This would be responsible for the relatively poor bacterial growth and energy metabolism when using ethanol as the sole carbon source.

To further confirm the mevalonic acid was synthesized from ethanol, deuterium-labeled ethanol-D6 was used as the feedstock instead of normal ethanol [28]. The total ion current chromatogram and mass spectrum of mevalonic acid lactone generated from ethanol-D6 were shown in Fig. 5b, d. The ion fragments of mevalonic acid lactone derived from ethanol or ethanol-D6 were analyzed [29]. Two major fragments of mevalonic acid lactone at m/z 43 and 71 were shifted to m/z 45 and 79 when ethanol-D6 was supplemented to the culture media. The molecular ion peak was increased by ten (from m/z 130 to 140). Evidences for partial incorporation of the deuterium atom would be seen from the ion fragments at m/z 43, 75 and 77, indicating that a proportion of the hydrogen in mevalonic acid might be from the initially added glucose.

## Discussion

Although *E. coli* can utilize a variety of feedstocks as the carbon sources, ethanol has rarely been tested. Ethanol can diffuse freely into and out of cell membranes. It can compromise the permeability barrier provided by cell membranes and have a certain toxic effect on cells [30]. *Escherichia coli* can adapt to ethanol by altering its membrane lipid composition. Genes responsible for ethanol tolerance including *gcv*, *betIBA*, *betT* and *marAB* were identified through transcriptome analysis [31]. Several ethanol-resistant mutants of *E. coli* with high-concentration ethanol resistance were isolated through random mutagenesis which had the potential for commercial ethanol production [32]. Considering that this study aims to investigate the utilization of ethanol by *E. coli*, the concentration of ethanol in the medium will not be too high. Moreover, we can use the feeding strategy to continuously add ethanol to the fermentation broth. Therefore, commonly used *E. coli* strains can fully meet the requirements of ethanol utilization.

Alcohol dehydrogenase catalyzes the rate-limiting step for ethanol catabolism in our engineered strain. Alcohol dehydrogenase can utilize either NAD^+^ or NADP^+^ as the electron donor. When using ethanol as the substrate, alcohol dehydrogenase is found to be NAD^+^ specific [33]. Ethanol has a high redox potential, which is not preferable for NAD^+^ reduction. According to the second law of thermodynamics, the changes of Gibbs free energy should be negative for the enzymatic reaction to easily proceed. Thermodynamic analysis of the reaction $(\text{Ethanol}\, + \,{\text{NAD}}^{ + } { \leftrightharpoons }{\text{Acetaldehyde}}\, + \,{\text{NADH}}\, + \,{\text{H}}^{ + } )$ shows that it has a positive standard Gibbs free energy change (ΔG_0_^′^) of 23.7 kJ mol^−1^ which is endergonic, indicating that it cannot be carried out efficiently from the perspective of thermodynamics [34]. Only if acetaldehyde was further and quickly metabolized to acetic acid, the utilization of ethanol could be realized from the point of view of thermodynamics. Acetaldehyde could be hardly detected in the fermentation processes, thus promoting the overall metabolic flux to the direction of ethanol utilization.

We tested different alcohol dehydrogenases and found that only AlcA from *A. nidulans* could efficiently oxidize ethanol. Enzymes can lower the activation energy (E_a_) of a reaction for the reactants to become products. The E_a_ barrier is different according to the substrates and their binding modes with the alcohol dehydrogenases [35]. Most alcohol dehydrogenases displayed a relatively high E_a_ value when using ethanol as the substrate [36]. The E_a_ barrier should be much lower in *A. nidulans* alcohol dehydrogenase than in other alcohol dehydrogenases for hydride transfer. Therefore, the engineered strain harboring this enzyme could efficiently utilize ethanol as the sole carbon source.

Ethanol can be used as the sole carbon source for cell growth and product biosynthesis in our engineered strains, but there is still a big gap in the growth rate compared with glucose as the carbon source. Glucose is the most commonly used carbon source in *E. coli*, which enters the central metabolic pathway through glycolysis [37]. The oxidation of glucose to acetyl-CoA generates four NADH and consumes one two ATP $(\text{G}{\text{lucose}}\, + \, 2 {\text{ CoA}}\, + \, 4 {\text{ NAD}}^{ + } \, + \, 2 {\text{ ADP}}\, + \, 2 {\text{ Pi}}{ \leftrightharpoons } 2 {\text{ acetyl}} - {\text{CoA}}\, + \, 2 {\text{ CO}}_{ 2} \, + \, 4 {\text{ NADH}}\, + \, 4 {\text{ H}}^{ + } \, + \, 2 {\text{ ATP}})$, while the oxidation of ethanol to acetyl-CoA generates two NADH and consumes one ATP $({\text{Ethanol}}\, + \,{\text{CoA}}\, + \, 2 {\text{ NAD}}^{ + } \, + \,{\text{ATP}}{ \leftrightharpoons } 2 {\text{ acetyl}} - {\text{CoA}}\, + \, 2 {\text{ NADH}}\, + \, 2 {\text{ H}}^{ + } \, + \,{\text{ADP}}\, + \,{\text{Pi}})$ . Although NADH can be further convert to ATP via the tricarboxylic acid (TCA) cycle, the lack of substrate level phosphorylation would significantly hamper the growth rate of the microorganism [38]. Therefore, ethanol cannot be utilized as efficiently as glucose. The study of mixed substrates will be direction for the utilization of ethanol as carbon source.

## Conclusions

In summary, an engineered *E. coli* which could utilize ethanol as the sole carbon source was constructed by heterologous expression of the alcohol dehydrogenase and aldehyde dehydrogenase from *A. nidulans*. The strain could metabolize ethanol for both cell growth and value-added chemical biosynthesis. Mevalonic acid production was achieved by further introduction of its biosynthetic pathway. Although the production of mevalonic acid was still very low, this study can serve as the basis for the construction of more robust strains for ethanol utilization in the future. Further work will be done to improve ethanol utilization by optimizing the metabolic pathways.

## Methods

### Enzymes, oligonucleotide primers, reagents and culture media

FastDigest restriction enzymes and T4 DNA ligase were purchased from Thermo Scientific (Pittsburgh, USA). PrimerSTAR Max Taq DNA polymerase was obtained from Takara (Dalian, China). The oligonucleotide primers used for plasmids construction were given in Table 1. The bacterial genomic DNA isolation kit, plasmid extraction kit and DNA gel extraction kit were offered by Omega Bio-tek (Norcross, USA). Mevalonic acid lactone was obtained from Acros Organics (Geel, Belgium). Deuterium-labeled ethanol-D6 was purchased from Aladdin (Shanghai, China). All other chemicals used in this study were of analytical grade. LB media (10 g L^−1^ tryptone, 5 g L^−1^ yeast extract, 10 g L^−1^ NaCl) were used for DNA manipulations. M9 media (Na_2_HPO_4_ 6 g L^−1^, KH_2_PO_4_ 3 g L^−1^, NH_4_Cl 1 g L^−1^, NaCl 0.5 g L^−1^) were used for shake-flask cultivations. Ampicillin (100 mg ml^−1^) or chloramphenicol (34 mg ml^−1^) or both of them were supplemented into the media if necessary.

**Table 1:** Primers used in this study for plasmids construction

Primers	Sequences
EcadhE_F_BamHI	CGCGGATCCGATGGCTGTTACTAATGTCGCTGAAC
EcadhE_R_XhoI	CCGCTCGAGTTAAGCGGATTTTTTCGCTTTTTTCTC
EcadhP_F_NcoI	CATGCCATGGATATGAAGGCTGCAGTTGTTACGAAGG
EcadhP_R_XhoI	CCGCTCGAGTTAGTGACGGAAATCAATCACCATG
EcaldA_F_NcoI	CATGCCATGGGCATGTCAGTACCCGTTCAAC
EcaldA_R_XhoI	CCGCTCGAGTTAAGACTGTAAATAAACCACCTGG
TrcEcaldA_F_XhoI	CCGCTCGAGCTGTTGACAATTAATCATCCGGC
TrcEcaldA_R_EcoRI	CCGGAATTCTTAAGACTGTAAATAAACCACCTGG
AnalcA_F_NcoI	CATGCCATGGATATGTGCATCCCGACCATGCAGTG
AnalcA_R_XhoI	CCGCTCGAGTTATTCCGGCATTTCCAGAACG
AnaldA_F_NcoI	CATGCCATGGATATGTCTGACCTGTTCACCACC
AnaldA_R_XhoI	CCGCTCGAGTTAAGCGAACAGAGCGTCACC
TrcAnaldA_F_PstI	AAAACTGCAGCTGTTGACAATTAATCATCCGGC
TrcAnaldA_R_SalI	ACGCGTCGACTTAAGCGAACAGAGCGTCACC
Ecacs_F_NcoI	CATGCCATGGGCATGAGCCAAATTCACAAACACACC
Ecacs_R_BamHI	CGCGGATCCTTACGATGGCATCGCGATAGCCTG

The restriction sites in the primers were underlined

### Cloning of genes and construction of recombinant plasmids

The bacterial strains and recombinant plasmids used in this study were listed in Table 2. The *adhE, adhP* and *aldA* genes of *E. coli* were amplified from genomic DNA and ligated into the pTrcHis2B vector, resulting pTrc-EcadhE, pTrc-EcadhP and pTrc-EcaldA. PCR reactions were performed using pTrc-EcaldA as the template and primers that allowed amplifying Trc promoter along with the *aldA* structure gene. Then the TrcEcaldA fragment was cloned into pTrc-EcadhE or pTrc-EcadhP to create pTrc-EcadhEaldA or pTrc-EcadhPaldA. The *alcA* and *aldA* genes from *A. nidulans* was optimized according to the codon usage table of *E. coli* using an online tool (www.jcat.com), chemically synthesized and cloned into pUC57 vector by BGI (Shenzhen, China). Then *alcA* and *aldA* were sub-cloned into the restriction sites *Nco*I/*Xho*I of vector pTrcHis2B, creating pTrc-AnalcA and pTrc-AnaldA. The recombinant plasmid pTrc-AnalcdA harboring both *alcA* and *aldA* was constructed using a similar strategy. The native *E. coli* acetyl-CoA synthetase (encoded by acs) was cloned into the expression vector pACYCDuet-1, resulting pA-Ecacs. The recombinant plasmid pA-EfmvaES harboring *Enterococcus faecalis mvaE* and *mvaS* (encoding the mevalonic acid biosynthesis pathway) was constructed in our previous work [39]. All recombinant plasmids listed in Table 2 were sequenced to verify the cloning accuracy.

**Table 2:** Strains and plasmids used in this study

Strains or plasmids	Genotype/Description	Sources
Strains		
*E. coli* DH5α	*F *^−^ *φ80lacZΔM15 Δ(lacZYA*-*argF)U169 recA1 endA1 hsdR17 (rK *^−^*, mK*^+^*) phoA supE44 λ *^−^ *thi*-*1 gyrA96 relA1*	Transgen Biotech
*E. coli* Top10	*F *^−^ *mcrA Δ(mrr*-*hsdRMS*-*mcrBC) φ80lacZΔM15 ΔlacX74 recA1 araD139 Δ(ara*-*leu)7697 galU galK λ *^−^ *rpsL(StrR) endA1 nupG*	Transgen Biotech
*E. coli* JM109	*F *^−^ *traD36 proA*^+^*B*^+^ *lacIq Δ(lacZ)M15/Δ(lac*-*proAB) glnV44 e14 *^−^ *gyrA96 recA1 relA1 endA1 thi hsdR17*	Transgen Biotech
*E. coli* BL21(DE3)	*F *^−^ *ompT hsdS*_*B*_ (r_B_^−^ m_B_^−^) *gal dcm rne131* (DE3)	Transgen Biotech
Plasmids		
pUC57	*Amp*^*r*^ *oripUC*	BGI
pTrcHis2B	*Amp*^*r*^ *oripBR322 lacI*^*q*^ *Trcp*	Invitrogen
pACYCDuet-1	*Cm*^*r*^ *orip15A lacI*^*q*^ *T7p*	Novagen
pTrc-EcadhE	pTrcHis2B harboring *E. coli adhE* gene	This study
pTrc-EcadhP	pTrcHis2B harboring *E. coli adhP* gene	This study
pTrc-EcaldA	pTrcHis2B harboring *E. coli aldA* gene	This study
pTrc-EcadhEaldA	pTrcHis2B harboring *E. coli adhE* and *aldA* gene	This study
pTrc-EcadhPaldA	pTrcHis2B harboring *E. coli adhP* and *aldA* gene	This study
pUC-AnalcA	pUC57 harboring *A. nidulans alcA* gene	This study
pUC-AnaldA	pUC57 harboring *A. nidulans aldA* gene	This study
pTrc-AnalcA	pTrcHis2B harboring *A. nidulans alcA* gene	This study
pTrc-AnaldA	pTrcHis2B harboring *A. nidulans aldA* gene	This study
pTrc-AnalcdA	pTrcHis2B harboring *A. nidulans alcA* and *aldA* genes	This study
pA-Ecacs	pACYCDuet-1 harboring *E. coli acs* gene	This study
pA-EfmvaES	pACYCDuet-1 harboring *E. faecalis mvaE* and *mvaS* genes	[39]

### Shake-flask fermentation

Shake-flask fermentation was performed in 250 ml Erlenmeyer flasks containing 50 ml M9 mineral media supplemented with glucose or ethanol. Single colonies of *E. coli* strains harboring different recombinant plasmids were picked from LB agar plate and inoculated into a test tube containing 3 ml liquid LB media. After overnight incubation, the seed culture was inoculated 1:100 into fresh M9 media and cultured at 37 °C. The optical density at 600 nm (OD_600_) of the culture was monitored and the bacterial cells were induced by 0.2 mM isopropyl β-d-thiogalactoside (IPTG) at early exponential stage. Then the cultivation temperature was shifted to 30 °C. After the initial carbon sources were exhausted, ethanol or ethanol-D6 was added to the culture broth. Samples were taken at appropriate intervals to determine OD_600_, residual glucose and ethanol, production of acetaldehyde, acetic acid and mevalonic acid during the whole fermentation process. The fermentation experiments were conducted in triplicate.

### Analytical methods

Cell density was determined by measuring the OD_600_ of the culture sample using a Varian Cary-50 UV–Vis spectrophotometer (Palo Alto, USA). The culture broth was diluted appropriately to bring down OD_600_ to the range of 0.2–0.8. After measurement, the value was multiplied by the dilution factor to achieve the actual OD_600_.

The concentration of residual glucose was quantified using a YSI 2950D Biochemistry Analyzer (Yellow Springs, USA). The cultures were centrifuged at 12,000 rpm for 1 min to obtain the supernatant. Samples were determined and compared with fresh M9 mineral media in triplicate using the YSI Biochemistry Analyzer.

Ethanol, acetaldehyde and acetic acid in the fermentation broth were determined by gas chromatography (GC). The fermentation supernatant was filtered through 0.22 μm membranes and then directly analyzed by GC. Agilent (Santa Clara, USA) 7890B GC system equipped with a flame ionization detector (FID) and a DB-FFAP column (30 m × 0.32 mm × 0.25 μm) was used for GC analysis. The injector temperature was 250 °C, the FID temperature was 250 °C and the split ratio was 1:10. The oven temperature was programmed as follows: initially held at 40 °C for 5 min, ramped at 20 °C min^−1^ to 240 °C and finally maintained at 240 °C for 3 min. High-purity nitrogen was used as the carrier gas with a flow rate of 1 ml min^−1^.

Mevalonic acid was identified by GC–MS as described previously [40]. Bacterial cultures were centrifuged to collect the supernatants. Then the samples were acidified to pH below 2 by the addition of HCI and incubated at 45 °C for 1 h to convert mevalonic acid to the lactone form. The mixture was saturated with anhydrous Na_2_SO_4_ and mevalonic acid lactone was extracted with ethyl acetate twice. The organic phase was further dried with anhydrous Na_2_SO_4_ and evaporated under nitrogen at room temperature. The dry samples were then dissolved in ethyl acetate and analyzed by an Agilent GC–MS system (7890A/5975C). The GC–MS system was equipped with an HP-5 ms column (30 m × 0.25 mm × 0.25 μm) and using helium as the carrier gas at a flow rate of 1 ml min^−1^. The column was initially held at 100 °C for 2 min followed by a gradient of 10 °C min^−1^ to 250 °C and then followed by a final hold at 250 °C for 5 min. The injector was used in the splitless mode at 250 °C. The EI ionizing voltage was 70 eV. Total ion current monitoring was performed at the mass range of m/z 35–300. The peak of mevalonic acid lactone was identified by the retention time of an external standard as well as comparing the mass spectrum with the National Institute of Standards and Technology (NIST) library.

## Data Availability

Not applicable.
